# How does cement choice affect screw stability in implant-supported restorations with different abutment angulations? An in vitro study

**DOI:** 10.1186/s12903-026-08503-3

**Published:** 2026-05-06

**Authors:** Shaza Bishti, Katarzyna Hubryj, Martin Homa, Stefan Wolfart, Taşkın Tuna

**Affiliations:** Department of Prosthodontics and Biomaterials, Centre for Implantology, Uniklinik RWTH Aachen, Pauwelsstrasse 30, Aachen, 52074 Germany

**Keywords:** Cement type, Implant restoration, Screw loosening, Abutment angulation

## Abstract

**Objective:**

To investigate the influence of cement type on the abutment-screw stability of single implant-supported restorations with different abutment angulations.

**Materials and methods:**

Fifty CAD/CAM implant-supported monolithic zirconia crowns simulating an upper premolar were divided into four groups (*n* = 10) according to the type of retention (SC: screwed, Cem: cemented), cement (TC: temporary, DC: definitve cement) and abutment angulation (SA: straight, AA: angulated abutment). Temp Bond NE and a self-adhesive resin cement (RelyX) were used for temporary and definitive cementation, respectively. All samples were subjected to dynamic loading with thermocycling in a chewing simulator (6336 cycles, 5 –55 °C, 98 N, 1.2 million cycles). Pre- and post-loading reverse torque values (RTVs) were measured using an electronic screwdriver, and the reverse torque differences (RTD) were thereafter calculated. The Kruskal-Wallis and Mann-Whitney tests were used for the statistical analysis.

**Results:**

Among test groups, the lowest RTD was seen in group Cem_AA_DC (2.12 ± 2.06Ncm), whereas the highest was found in group Cem_SA_TC (3.16 ± 0.18 Ncm). Statistically significant differences were seen between the screwed (SC_SA) and all cemented groups except Cem_SA_TC (*p* = 0.089). Among the cemented groups, no significant difference was observed between straight and angulated abutments, nor between temporary and permanent cement types.

**Conclusion:**

Definitive cementation, particularly in angulated abutments, was associated with higher torque retention compared to screw-retained or temporary cemented restorations.

**Clinical implication:**

Definitive cementation may contribute to improved screw stability of implant restorations, especially in angulated abutments, compared with temporary or screw-retained designs, highlighting the potential impact of retention method and cement choice on long-term mechanical performance.

## Introduction

Implant-supported restorations are widely recognized as a reliable treatment modality for replacing missing teeth, with reported long-term success and survival rates exceeding 90% [1–3]. However, despite their predictability, biological and technical complications are common, with screw loosening being among the most frequently reported technical complication [4–6]. The method of retention - whether screw- or cement-retained, plays a role in the incidence of screw loosening [5, 7, 8]. While screw-retained restorations allow easier access for retightening, they are more susceptible to preload loss over time [9–11]. In contrast, cement-retained restorations may demonstrate improved torque stability, possibly due to a more favorable distribution of occlusal forces and reduced micro-movement at the implant-abutment interface [9].

Cemented implant-supported restorations remain a widely and routinely used option in prosthetic dentistry, due to their favorable esthetics, passive fit, simplified handling and cost-effectiveness [12, 13]. However, a key drawback of these restorations is the inability to retain and subsequently retrieve the superstructure in a reliable way, when required for routine care and maintenance [13]. The retention of cemented restorations is influenced by multiple factors such as abutment geometry, surface treatment, cement space and the type of luting agent used [14–16]. The selection of an appropriate cement is therefore critical to achieve sufficient retention and predictable retrievability in cemented implant restorations [15, 17–20]. In clinical practice, temporary, semipermanent and permanent cements are routinely used, with each offering distinct advantages and disadvantages [13, 21, 22]. Several studies have investigated the retention forces of implant-supported restorations luted with different cements. It has been previously reported that a cement should have a retention force of 50–200 N to achieve the balance between sufficient retention under masticatory forces and retrievability whenever necessary without restoration damage [23, 24]. However, a recent study investigating the pull-off forces of implant-supported restorations with varying designs and surface roughnesses reported that a cement exhibiting a retention force of 20–60 N after clinical use can provide sufficient retention and allow predictable retrievability, regardless of which cement is used, and after putting other factors such as abutment taper, height and width, cement film thickness and surface roughness into consideration [25]. This suggests that, in cases where abutment height is insufficient, it may be necessary to choose a cement with a higher retention force, potentially requiring the substitution of a temporary cement with a permanent one.

Due to anatomical limitations such as insufficient bone volume or unfavorable ridge morphology, implants are not always placed in ideal axial positions. To compensate for such suboptimal implant angulation, angulated abutments are commonly used [26]. These abutments help redirect the prosthetic axis to achieve better functional and esthetic outcomes. However, they may influence stress distribution at the implant-abutment interface and potentially affect screw joint stability [27, 28]. Some in-vitro studies have reported increased stress concentrations and micromovements associated with angulated abutments, which could contribute to mechanical complications such as screw loosening over time [9, 27–29]. However, clinical studies remain inconclusive, and the impact of angulation appears to be multifactorial, depending on prosthetic design, loading conditions, and material choices [30, 31].

The influence of different cement types—whether temporary, semi-permanent, or permanent—on the retention and clinical success of implant restorations, as well as their potential impact on biological and technical complications, has been thoroughly investigated in the literature [32–35]. However, the relationship between cement type and screw loosening has received limited attention in current research. In particular, few studies have explored the interaction between cement type and prosthetic design variables, such as abutment angulation, under controlled conditions. Therefore, the aim of this in-vitro study is to investigate the influence of cement type on the screw stability of single implant-supported restorations with a specific implant system and selected abutment angulations. The null hypotheses set are: (1) there is no significant difference between temporarily and definitively cemented restorations in terms of screw loosening, and (2) abutment angulation has no influence on screw stability of implant-supported restorations under the conditions tested.

## Materials and methods

**Fig. 1:**
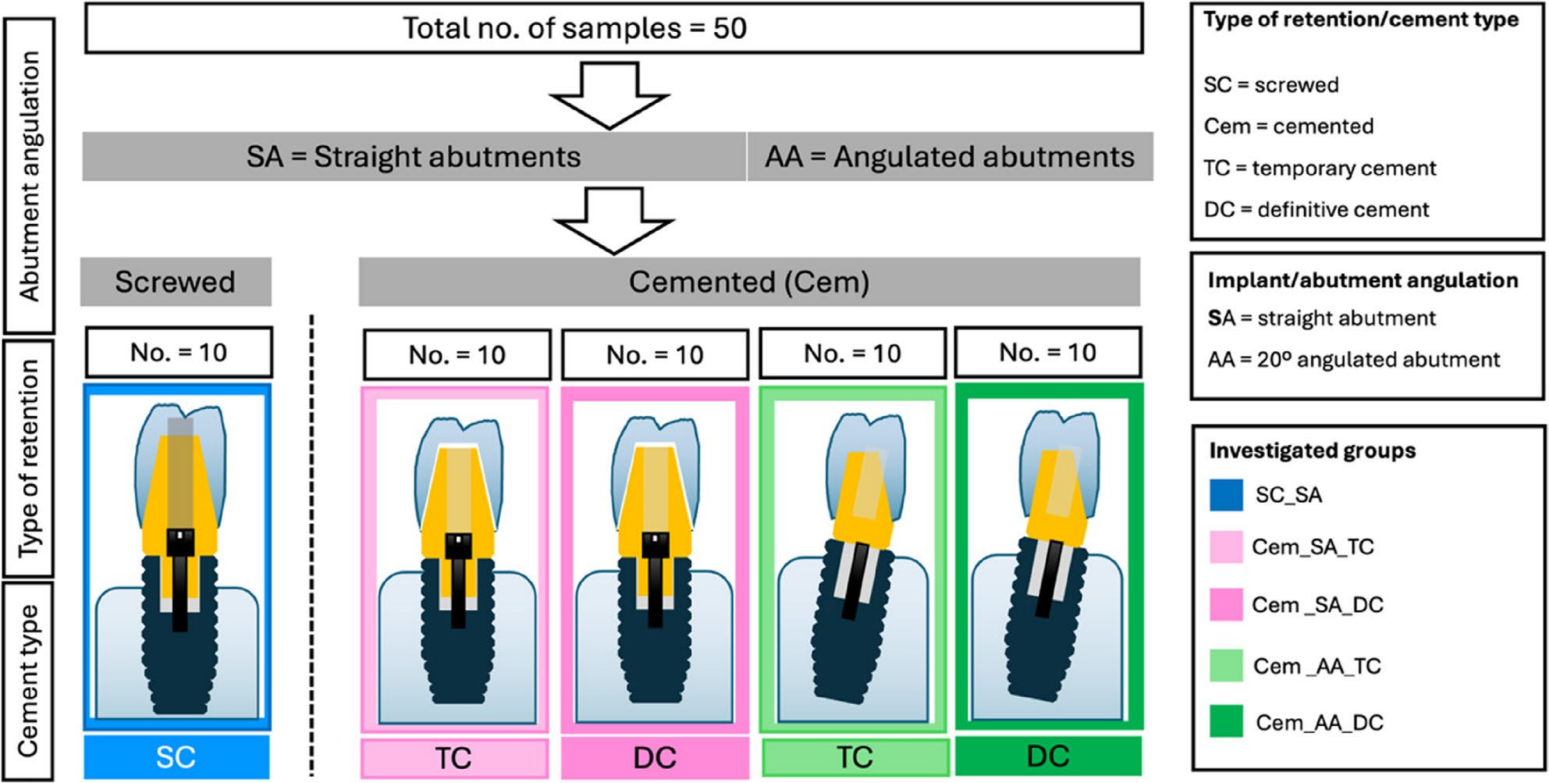
Schematic image showing the five test groups. SC_SA: Screwed restorations with straight abutments, Cem_SA_TC: Cemented restorations with straight abutments and temporary cementation, Cem_SA_DC: Cemented restorations with straight abutments and definitive cementation, Cem_AA_TC: Cemented restorations with angulated abutments and temporary cementation, Cem_AA_DC: Cemented restorations with angulated abutments and definitive cementation

A total number of 50 dental implants (length = 13 mm, ⌀ = 4.3 mm; Promote Plus, CAMLOG Biotechnologies AG, Basel, Switzerland) were used for the investigation. The implants were embedded in sample holders filled with autopolymerizing acrylic resin (PalaXpress, Kulzer GmbH, Hanau, Germany) using a custom-made fixture. This fixture allows for adjustable alignment along the horizontal and vertical axes, as well as angulation, enabling standardized implant placement. To simulate marginal bone loss in bone-level implants, the implant shoulders were positioned 2 mm above the resin level, in accordance with DIN ISO 14,801. The samples were divided into five groups (*n* = 10) according to the type of retention, cement and implant/abutment angulation (Table 1; Fig. 1). Sample size determination was based on previously published data with a comparable experimental design [9]. Using an observed effect size of approximately 1.2 for reverse torque difference between groups and a significance level of α = 0.05, the calculated statistical power exceeded 80%, confirming the adequacy of 10 specimens per group.

**Table 1:** Investigated groups according to type of retention, cement type and implant/abutment angulation

Group	Type of retention	Implant/abutment anglulation	Type of abutment/brand	Cement type
SC_SA	screwed	straight	Titanium base CAD/CAM abutments, Camlog Biotechnologies AG	NA
Cem_SA_TC	cemented	straight	Esthomic Abutment, gingival height 1–1.8 mm, Camlog Biotechnologies AG	Temporary cement
Cem_SA_DC	cemented	straight	Esthomic Abutment, gingival height 1–1.8 mm, Camlog Biotechnologies AG	Definitive cement
Cem_AA_TC	cemented	Angulated (20°)	Esthomic Abutment 20°, gingival height 1–1.8 mm, Type A, Camlog Biotechnologies AG	Temporary cement
Cem_AA_DC	cemented	Angulated (20°)	Esthomic Abutment 20°, gingival height 1–1.8 mm, Type A, Camlog Biotechnologies AG	Definitive cement

*SC* screwed, *Cem* cemented, *SA* straight abutment, *AA* angulated abutment, *TC* temporary cement, *DC* definitive cement, *NA* not applicable

For the restorations, a total of 50 CAD/CAM monolithic zirconia crowns simulating an upper premolar were fabricated (3Y-TZP, Zirlux FC2, Henry Schein, Langen, Germany). The crowns were designed using 3Shape Dental Designer software (version 2013/2014; 3Shape A/S, Copenhagen, Denmark). The cement space was set to 60 μm, whereas the minimum wall thickness was defined as 0.5 mm. The CAD designs were then processed the CAM software (Zenotec^→^ CAM software, Wieland Dental, Pforzheim, Germany) and subsequently milled using a milling unit (Zenotec^Ⓡ^ 1 milling unit, Wieland Dental, Pforzheim, Germany).

**Fig. 2:**
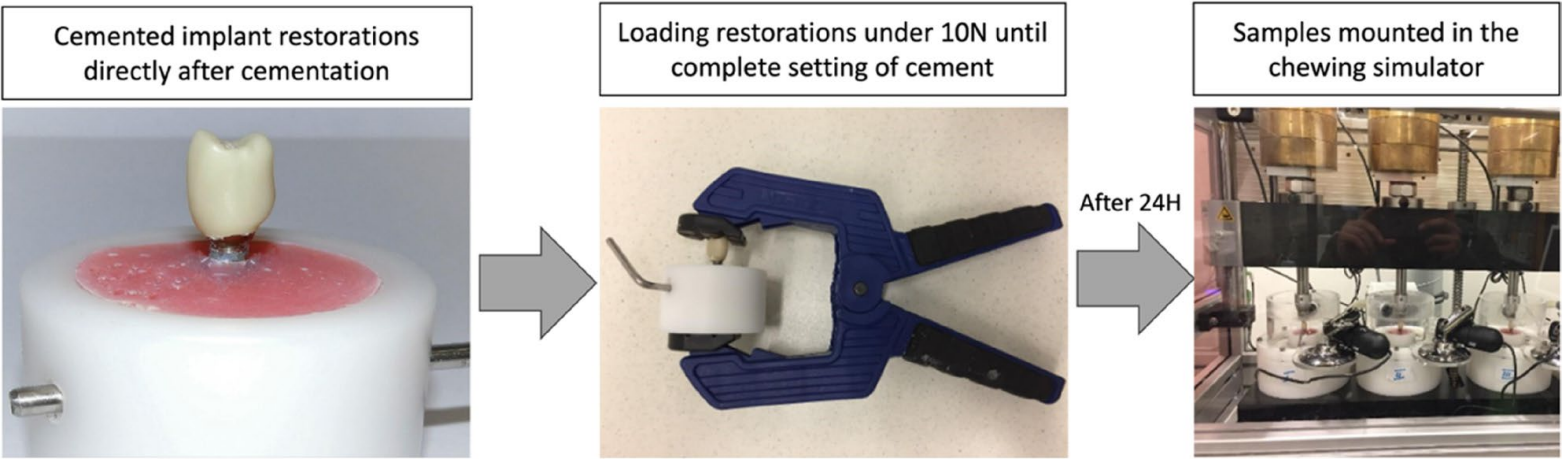
A tested sample being cemented and loaded under 10 N until complete setting of cement. After 24 h, samples were loaded in a dual-axis chewing simulator. Steatite balls were used as anatgonists to deliver the vertical and lateral forces

The screw-retained group (SC_SA) received titanium base CAD/CAM abutments (Camlog Biotechnologies AG, Basel, Switzerland) with straight configuration. In contrast, all cement-retained groups received prefabricated titanium Esthomic abutments (Camlog Biotechnologies AG, Basel, Switzerland). Straight abutments (gingival height 1–1.8 mm) were used in groups Cem_SA_TC and Cem_SA_DC, whereas 20° angulated abutments (Esthomic Abutment 20°, Type A, gingival height 1–1.8 mm) were used in groups Cem_AA_TC and Cem_AA_DC. The abutments were screwed to their respective implants with a torque of 20Ncm according to manufacturer’s instructions. After 10 min, all abutments were retightened by the same operator using the same torque value to avoid the settling effect. The inner surface of each crown was air-abraded using 50 μm aluminum oxide at a pressure of 1 bar for 15s at a standardized distance of 10 mm. The nozzle was maintained perpendicular to the internal surface throughout the procedure, and all specimens were treated by a single operator to ensure consistency. Pressure was controlled using the built-in regulator of the air-abrasion unit. Subsequently, all crowns were ultrasonically cleaned in 70% alcohol (Renfert Easyclean, Hilzingen, Germany) for 5 min and then air-dried. Crowns in groups Cem_SA_TC and Cem_AA_TC were cemented using a temporary cement (TempBond NE, Kerr Dental, Kloten, Switzerland), whereas Cem_SA_TC and Cem_AA_TC were cemented with a self-adhesive resin cement (RelyX Unicem 2, 3 M Espe, Seefeld, Germany). Both cements were mixed according to manufacturer’s instructions then applied evenly to the inner surface of the crowns. The crowns were seated onto their corresponding abutments with finger pressure. After removal of excess cement, the whole sample was subjected to a 10 N load until the cement was completely set. Following cementation, all samples were stored for 24 h before undergoing chewing simulation (Fig. 2).

For group SC_SA, crowns were luted to their corresponding titanium base CAD/CAM abutments (Camlog Biotechnologies AG, Basel, Switzerland) using a self-curing luting composite (Multilink Hybrid abutment cement, Ivoclar Vivadent, Schaan, Liechtenstein). The crown-abutment complex was subsequently screwed to their implants with a torque of 20Ncm according to manufacturer’s instructions, then retightened after 10 min. The screw access channels were sealed using a foam pellet and a light-curing composite resin (Tetric EvoCeram, Ivoclar Vivadent, Pforzheim, Germany).

All implant restorations were mounted in a dual-axis chewing simulator (CS-4.8, SD Mechatronik, Feldkirchen, Westerham, Germany). Steatite ceramic balls (6 mm diameter) served as antagonists and were attached to the upper sample holders to deliver vertical and lateral forces to the restorations (Fig. 2). The samples underwent simultaneous thermocycling (6336 cycles, 5 –55 °C, with a dwell time of 60 s) and mechanical loading at 98 N for a total of 1.2 million cycles. Based on previous reports estimating approximately 240,000–250,000 chewing cycles per year, this protocol simulates nearly five years of clinical function in the oral environment [36, 37]. The chewing simulator was operated in its 8-chamber configuration. Due to the total number of specimens (*n* = 50), thermomechanical aging was performed in consecutive batches. All specimens were stored under standardized laboratory conditions for 24 h after cementation prior to loading, and reverse torque measurements were performed immediately after completion of the loading protocol. No prolonged storage periods occurred between cementation, aging, and torque evaluation, thereby ensuring identical environmental conditions for all specimens.

For torque measurements, an occlusal access hole was created in the cemented groups (Cem_SA_TC, Cem_AA_TC, Cem_SA_DC and Cem_AA_DC) using a standardized protocol under low-speed drilling with water cooling, performed by the same operator using the same drill system for all specimens to minimize heat generation and vibration. Then, the foam pellets were carefully removed to expose the screws. In group SC_SA, both the composite sealing material and the foam pellets were removed to access the abutment screws. Each specimen was then securely positioned, and the reverse torque value (RTV)—the torque required to loosen the abutment screw—was performed by the same operator using a digital torque controller (200 cNm, Holex, Germany). according to the manufacturer’s instructions. The device was verified for proper function prior to the experiment to ensure consistent and reproducible torque application. Subsequently, the reverse torque difference (RTD) for each sample was calculated using the formula: RTD (Ncm) = Preloading screw-in torque − Postloading screw-out torque (RTV).

The statistical analyses were performed using IBM SPSS Statistics (Version 29.0.0, IBM Corp., Armonk, NY, USA). The primary outcome variable was the removal torque difference (RTD), representing the change in preload after aging. Removal torque values (RTV) were recorded to calculate RTD. Normality of the data within each group was assessed using the Shapiro–Wilk and test. Homogeneity of variances was evaluated using Levene’s test. Due to violations of the assumptions for parametric testing (i.e., non-normal distribution and unequal variances in some groups), a non-parametric approach was chosen. A Kruskal–Wallis test was used to compare RTV among the five experimental groups, defined by combinations of retention type (screw-retained, temporarily cemented, definitively cemented) and abutment angulation (0° or 20°). When the Kruskal–Wallis test indicated a statistically significant difference, pairwise comparisons between groups were conducted using the Mann–Whitney-U test. To control for multiple comparisons, the Holm–Bonferroni method was applied to adjust p-values. Statistical significance was set at *p* < 0.05.

## Results

Normality of the data was assessed using the Shapiro–Wilk test. The screw-retained (W = 0.955, *p* = 0.726) and temporary cemented groups (W = 0.941, *p* = 0.245) demonstrated normal distribution, whereas the definitive cemented group showed significant deviation from normality (W = 0.832, *p* = 0.003). Homogeneity of variances was evaluated using Levene’s test, which revealed significant differences among groups F = 2.915, *p* = 0.032). As assumptions of normality and homogeneity were not fully met, non-parametric tests (Kruskal–Wallis and Mann–Whitney U tests) were used for subsequent analyses.

All investigated samples exhibited a measurable degree of torque loss following dynamic loading, as indicated by the recorded reverse torque difference values (RTDs). Table 2 summarizes the mean RTDs and corresponding standard deviations across the five experimental groups.

**Table 2:** Mean values and standard deviations of the reverse torque difference between pre- and postloading torque values among the five investigated groups

Group	Preloading screw-in torque (Ncm)	Post-loading screw-out torque (Ncm)	Reverse torque difference (RTD ± SD)
SC_SA	20.11 ± 0.16	15.46 ± 1.76	4.65 ± 1.79
Cem_SA_TC	20.47 ± 0.26	17.31 ± 0.75	3.16 ± 0.81
Cem_SA_DC	20.34 ± 0.31	17.81 ± 0.71	2.56 ± 0.82
Cem_AA_TC	20.28 ± 0.22	17.29 ± 0.91	2.99 ± 0.87
Cem_AA_DC	20.46 ± 0.27	18.34 ± 2.17	2.12 ± 2.06

*SC* screwed, *Cem* cemented, *SA* straight abutment, *AA* angulated abutment, *TC* temporary cemented, *DC* definitive cemented, *RTD* reverse torque difference, *SD* standard deviation

**Fig. 3:**
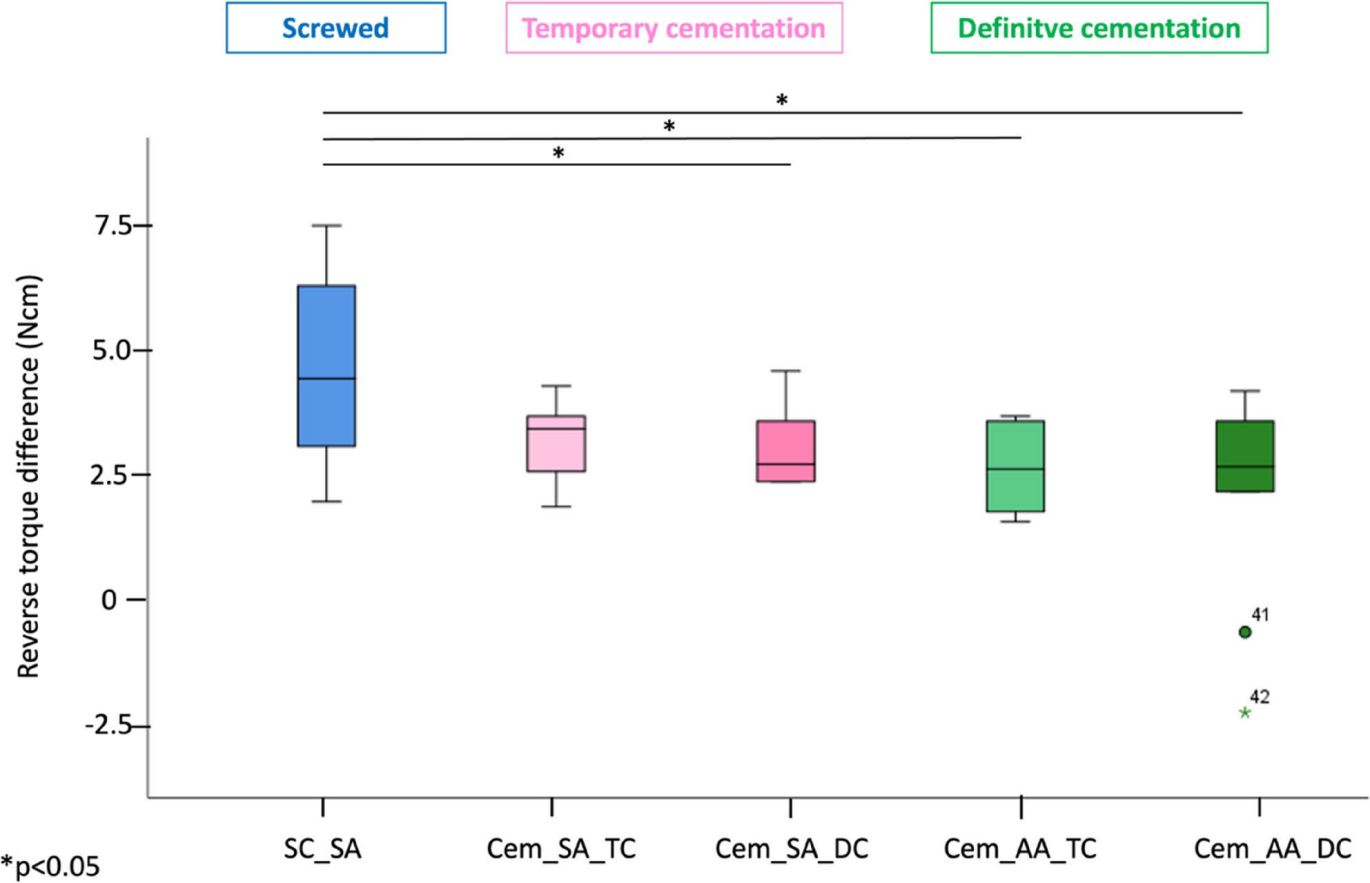
Boxplots representing the RTD values among the five investigated groups (SC_SA, Cem_SA_TC, Cem_SA_DC, Cem_AA_TC and Cem_AA_DC). (*) indicate significant differences between the groups

The lowest reverse torque difference was seen in group Cem_AA_DC with a RTD value of 2.12 ± 2.06Ncm, whereas the highest RTD was found in the screw-retained group (4.65 ± 1.79Ncm, SC_SA). Statistical analysis showed significantly lower RTVs in the definitively cemented groups (Cem_SA_DC and Cem_AA_DC) compared to the screw-retained group. A significant reduction in RTVs was also observed in the temporarily cemented group with angulated abutments (Cem_AA_TC). However, no significant difference was found between the screwed group and the temporarily cemented group with straight abutments (Cem_SA_TC), suggesting that the observed decrease in RTV may be associated with the combined effect of temporary cementation and increased abutment angulation. However, the Cem_AA_DC group demonstrated a relatively high standard deviation compared to its mean value, indicating considerable variability within this group (Fig. 3).

**Fig. 4:**
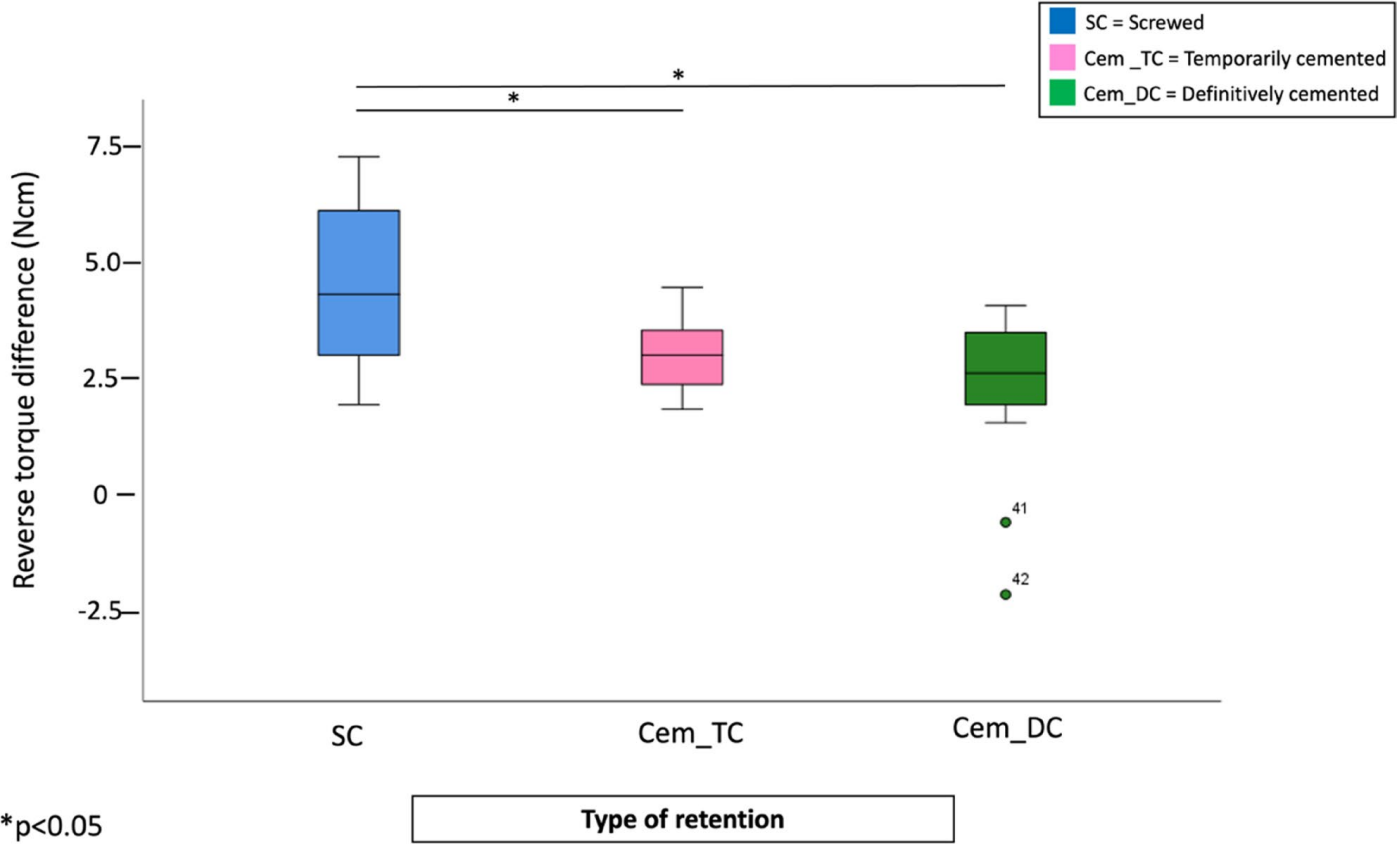
Boxplots representing the RTD values according to retention/cement type, regardless of implant/abutment angulation. (*) represent significant differences between the groups

Regardless of abutment angulation, the screwed group (SC_SA) showed significantly higher reverse torque differences values compared to both definitive and temporary cemented groups (*p* = 0.004, *p* = 0.023, and *p* = 0.019, respectively). No significant differences were observed between the temporary and definitive cemented groups (Fig. 4).

**Fig. 5:**
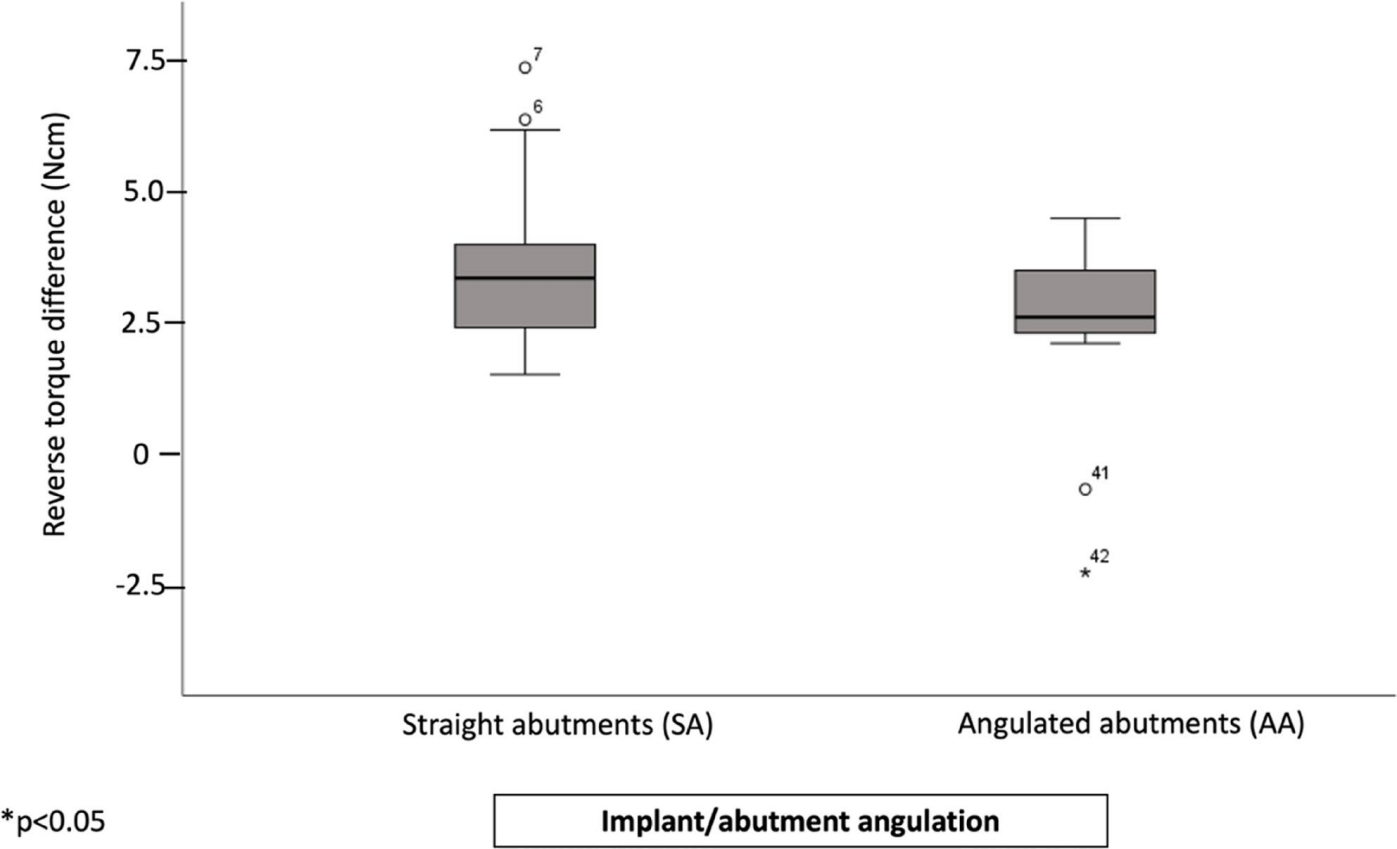
Boxplots representing the RTD values according to implant/abutment angulation, regardless of. retention/cement type. No significant differences between the groups were observed

Investigating the influence of abutment angulation, regardless of the cement type showed no significant differences between straight and angulated abutments (Fig. 5).

## Discussion

This in-vitro study aimed to investigate the influence of cement type on the screw stability of implant-supported restorations with different abutment angulations after 5 years of artificial aging. While retention and abutment angulation have been individually investigated in previous studies, the influence of cement type and the combined effect of these different factors on post-loading torque loss has received comparatively less attention in the literature.

Our findings revealed that screw-retained restorations exhibited significantly higher torque loss compared to cement-retained samples (RTD = 4.65 ± 1.79Ncm), particularly when compared with definitively cemented restorations with angulated abutments (group Cem_AA_DC) which demonstrated the lowest reverse torque difference (RTD = 2.12 ± 2.06Ncm). This outcome aligns with previous literature suggesting that screw-retained prostheses are more susceptible to preload loss over time, potentially due to microgaps, settling effects, and limited frictional resistance at the implant-abutment interface [9, 38, 39]. A methodological aspect that should be considered is the use of different abutment designs between the screw-retained and cement-retained groups. The screw-retained restorations were fabricated on titanium base CAD/CAM abutments, whereas the cement-retained restorations were placed on prefabricated straight or angulated Esthomic abutments. This approach was intentionally chosen to reflect common clinical workflows. However, differences in abutment geometry and implant–abutment interface characteristics may have influenced preload behavior and torque loss. Therefore, the observed differences cannot be attributed exclusively to the retention concept alone.

Among the cemented groups, no significant difference was observed between straight and angulated abutments, nor between temporary and permanent cement types. This finding suggests that, under loading conditions, abutment angulation did not adversely influence screw stability. Furthermore, although definitive cement groups demonstrated lower mean RTD values, the absence of statistical significance between cement types indicates that cementation itself may play a more substantial role in preload preservation than the specific cement formulation. These results imply that the presence of a cement layer may contribute to improved load distribution at the prosthetic interface, regardless of abutment geometry [9, 40]. Nevertheless, both definitively cemented groups demonstrated significantly lower reverse torque differences compared to the screw-retained group, suggesting that definitive cementation may enhance torque maintenance relative to screw retention alone. The lowest mean RTD was observed in the Cem_AA_DC group. This may be explained by the increased rigidity provided by resin cementation, which likely reduced micromovement at the crown–abutment interface during cyclic loading and thereby minimized preload loss. Under axial dynamic loading, the angulated abutment may also have influenced internal stress distribution within the prosthetic complex. However, given the relatively high variability within this group, the precision of this estimate is limited, and the finding should therefore be interpreted cautiously.

Although the current findings as well as other studies have reported greater torque loss in screw-retained restorations compared with cement-retained designs [9, 38, 39], other studies have demonstrated no significant differences between retention concepts [5, 10, 13]. Discrepancies may be attributed to variations in implant–abutment connection design, loading conditions (axial versus non-axial), abutment geometry, cement type, and aging protocols. Therefore, the influence of retention type on screw stability appears to be multifactorial rather than solely dependent on the retention mechanism itself.

Clinically, these findings have important implications. In scenarios where retrievability is not a primary concern, definitive cementation—particularly in angulated abutments—may provide superior mechanical stability and reduce the risk of abutment screw loosening. Cement-retained restorations are widely used and can provide excellent clinical outcomes when appropriate cementation protocols are followed. Nevertheless, previous studies have reported that residual excess cement, particularly from resin- or methacrylate-based definitive cements, may contribute to peri-implant tissue inflammation if not completely removed [41–44]. The position of the restoration margin and meticulous removal of excess cement are therefore critical for long-term biological success [45, 46]. Accordingly, while definitive cements may enhance mechanical performance, careful clinical handling and case selection remain essential. In contrast, screw-retained restorations offer advantages in retrievability and maintenance but may require monitoring of preload loss and occasional retightening.

The main limitations of the current study are the in-vitro conditions, which do not fully replicate the complexities of the oral environment. Biological responses such as peri-implant behaviour, variable occlusal loading, moisture and interpatient variability encountered in clinical setting may not be reflected. Another limitation is that only straight screw-retained abutments were tested. Angulated screw channel restorations were not included, although they are increasingly being used recently to manage implant misalignment while preserving screw retention benefits. Previous studies have shown that increased screw channel angulation can significantly reduce reverse torque values, likely due to suboptimal torque transmission and increased micromovement at the implant–abutment interface [47–50]. Hu et al. reported that as the angulation increased from 0° to 20°, the reverse torque values progressively decreased, with values dropping from 31.16 to 30.08 Ncm when a target torque of 35 Ncm was applied [48]. Similarly, El-Sheikh et al. reported that abutments with greater angulation exhibited lower torque retention after dynamic loading [47], emphasizing the importance of adjusting torque values based on abutment angulation to minimize the risk of screw loosening [47, 51]. Therefore, the torque stability of angulated screw-retained restorations remains a critical topic for future research. A further limitation of this study is that the screw-retained group involved luting of the crown-abutment complex prior to implant insertion, creating a hybrid interface. Consequently, the findings for this group may not fully reflect the behavior of purely screw-retained restorations, and caution should be used when generalizing these results to standard screw-retained crowns. In the cement-retained groups, occlusal access openings were created after thermomechanical aging to allow measurement of reverse torque values. This approach was intentionally chosen in order to simulate true cement-retained restorations during loading, without the presence of a screw-access channel that could alter stress distribution or cement behavior. Although post-aging drilling may theoretically induce microstructural alterations or surface defects in zirconia, all cemented specimens were treated identically under standardized conditions, ensuring consistency across groups. The access preparation was confined to the occlusal surface and performed with controlled parameters, minimizing the likelihood of clinically relevant crack propagation. Nevertheless, a potential influence on mechanical behavior cannot be completely excluded and should be considered when interpreting the results.

From a clinical perspective, the present findings suggest that cement-retained restorations may provide improved preload preservation compared with screw-retained restorations under axial functional loading. Therefore, in cases where screw access positioning compromises esthetics or occlusal design, cementation may represent a reliable alternative without negatively affecting screw stability. The absence of significant differences between temporary and definitive cement types indicates that the choice of cement should be guided primarily by retrievability requirements and biological considerations rather than mechanical performance alone. Temporary cements may be preferred in situations where future retrievability is anticipated, whereas definitive resin cements may be selected when enhanced retention is desired and careful cementation protocols can be ensured. Nevertheless, screw-retained restorations remain advantageous in cases requiring predictable retrievability, maintenance access, or when the risk of excess cement cannot be adequately controlled. Therefore, the choice between cement and screw retention should be based on comprehensive clinical evaluation rather than mechanical performance alone.

The above-mentioned limitations limit the generalizability of the results and suggest that the headline comparisons should be interpreted cautiously, as they may not fully reflect clinical behavior across all implant systems and prosthetic designs. Therefore, to validate and extend findings of the current study, further long-term in-vivo studies are needed to assess the clinical performance and retrievability of different retention systems and cement types under dynamic and biologically relevant conditions.

## Conclusion

Within the limitations of this in-vitro study, the findings suggest that definitive cementation, particularly in angulated abutments, may be associated with higher torque retention compared to screw-retained or temporary cemented restorations. These results emphasize the importance of carefully selecting the retention method and cement type based on individual clinical scenarios to optimize the mechanical longevity of implant-supported restorations.

## Data Availability

All data supporting the findings of this study are stored by the corresponding author. These data are available from the corresponding author upon reasonable request.
